# An Historical Essay on the Progress of the Medical Sciences, in Great Britain and on the Continent, during the Last Six Months of 1821

**Published:** 1822-01

**Authors:** 


THE LONDON
### Medical and Physical Journal.

1 OF VOL. XLVII.] JANUARY, 1822. [NO. 275.

"For many fortunate discoveries in medicine, and for the detection of numerous "errors, the world is indebted to the rapid circulation of Monthly Journals; "and there never existed any work to which the Faculty in EUROPE and "AMERICA were under deeper obligations than to the Medical and Physical "Journal of London, now forming a long, but an invaluable, series."—RUSH.

### AN HISTORICAL ESSAY
ON
### THE PROGRESS OF THE MEDICAL SCIENCES,
### IN GREAT BRITAIN AND ON THE CONTINENT,
*During the last Six Months of* 1821.

BY THE EDITOR.

### INTRODUCTION.

THE traveller, who is journeying onwards in search of an ill-defined, perhaps imaginary, place of rest, whither have tended all his toils, his exertions, and perseverance, will, ever and anon, check his career, and, casting an inquiring eye behind him, mark the progress he has made in his laborious, and often intricate, course. Thus, he that travels within the extended boundaries of medical science, endeavouring to descry those hidden and distant truths which are to reward his labours, must often pause in his inquiries, and, surveying what he has left, try to fix his own relative position, in order to advance with better chance of success.

It is precisely with a view to encourage these moments of reflection, which serve to facilitate our future researches, that we, as the monthly recorders of new facts and discoveries, think it our duty to pause at the termination of each half-yearly period, —in order that, by arranging, in a concentrated form, the stores of medical literature which accumulate all around us in rapid succession, we may be better able to retain what we have already learned; while we shall find all ulterior knowledge more easy of acquirement.

Our worthy predecessor, whose matchless activity and great erudition have been conspicuously displayed in the several

*Proëmia* with which he used to introduce each half-yearly volume, during the last three years, has left us an example worthy of imitation. Unfortunately, our occupations will not permit us to take a range so extensive, as the Proëmia embraced, in the circle of medical science. We must be satisfied with a more slender report of what has fallen under our notice during the preceding half-year; and, in default of proper abilities to support the character of historians, our readers will forgive us if we assume the more modest garb of simple chroniclers.

### I. GENERAL, DESCRIPTIVE, PATHOLOGICAL, AND COMPARATIVE ANATOMY.

It can scarcely be expected that we should have to record any new discovery, or many important and hitherto-unknown facts, in these various branches of zoological inquiry. The structure of man, as far as its mechanism is concerned, is well known. Each distinct organ has been diligently examined, and most accurately described. The effects of disease on these, separately as well as collectively, have been noted with a precision which, in our days, has served to give a more dignified character to the medical profession. The organization of inferior animals, even, has not escaped the attention of the anatomist; and, where obscurity prevailed in the pursuits of anthropotomy, light has been borrowed from the result of comparative dissections. One would be led to imagine that, after a long series of years spent in the closest and most attentive study of animal life, the mind of man had need of no further inquiry to comprehend the complicated machinery of the human fabric. That facts, however, warrant not such an induction, our readers, who have diligently followed the periodical advancement of anatomical science, will readily believe; and we need only refer to the anatomical productions of various nations during the last six months, to prove that, great as our stock of anatomical knowledge may be, what is already known of the structure of animals can scarcely equal what yet remains to be learned.

One of the most striking appearances which occasionally present themselves to the anatomist, is the transposition of all, or of some, of the viscera. Two instances of this deviation from the ordinary arrangement of parts have occurred during the period which our present Essay embraces: the one to Dr. CAMPBELL,* in the case of a child six weeks old, in the abdominal cavity of which were found only the liver and gall-bladder, the cardiac portion of the stomach, the termination of the colon, and the kidneys, covered with the peritoneum; while

* *Edinburgh Medical and Surgical Journal*, No. 69, p. 513.

the cavity of the thorax contained the rest of the stomach, the small intestines, and the remainder of the colon, with the spleen, pancreas, and great omentum :—and the other case to Mr. DESRUELLES, of Paris, in a French soldier, in whom the transposition of the thoracic and abdominal viscera was general.* Malformations of another kind are not unfrequently observed; and Mr. WINDSOR† has given the description of a fetus, in which the urinary bladder was found attached, throughout its whole length, to the abdominal parietes, the attachment extending for the space of six inches, and as far as the umbilicus. The author of the present Essay had, not long ago, an opportunity of dissecting the body of a child, having a spina bifida, in which the bladder presented exactly the same appearances as those described by Mr. Windsor; a circumstance which gave to the abdomen the character of ascites during life. The child lived three weeks.

Some recent anatomical inquiries into the pathology of the brain have been published by Mr. LALLEMAND and Dr. PINEL, jun.‡ Mr. SERRES, to whom we are indebted for the best work on the comparative anatomy of the brain, has also read, before the Society of Emulation in Paris, an anatomical theory of animal monstrosities.§ There is considerable ingenuity in his theory; and we were much pleased with the classification he has given of monstrosities dependant on excess or deficiency in the sanguiferous system.

The principal didactic books in anatomy published during the last half-year, are those of HIPP. CLOQUET,‖ and of his brother JULES CLOQUET.¶ The former, however, is only a new edition, and is mentioned in this place on account of the numerous additions that have been made to it. The second may be considered as an ingenious attempt to render the art of lithography subservient to the purposes of anatomical illustration. The author offers merely some general observations on the structure of the human fabric, as the vehicle of very accurate and really beautiful graphic representations of its different parts. The work is published in folio Numbers, a few of which have already made their appearance.

Few books have influenced the progress of medical studies more than the General Anatomy of BICHAT. It is, unquestionably, his best and principal work; but, excellent as

* *Bulletins de la Société Médicale d'Emulation.*

† *Edinburgh Medical and Surgical Journal*, No. 69, p. 563.

‡ *Lettres sur l'Encephale.* Montpellier.—*Recherches d'Anatomie sur les Alteration de l'Encephale.* Paris.

§ *Revue Medicale.* Octobre 1821.

‖ *Traité d'Anatomie Descriptive.* Par HIPP. CLOQUET. 2d edit.

¶ *Anatomie de l'Homme, ou Description et Figures Lithographiées de toutes les Parties du Corps Humain.*

that work may have hitherto appeared, the many additions made, since its publication, to the stores of anatomical knowledge, rendered it in some degree imperfect. A new edition being called for, Mr. BECLARD, whose anatomical knowledge needs no eulogium from us, has undertaken to revise it, and to add to it all those facts which the subsequent inquiries of anatomists have brought to light. To those who possess the first edition of Bichat's work in four volumes, the accommodation of a fifth volume has been offered by the editor, in which all the additions, notes, and corrections, have been included.* A new part of the *Encyclopèdie Methodique* has also been recently published, containing a continuation of the *Systême Anatomique*, written, principally, by Mr. HIPPOLITUS CLOQUET.

Two curious papers on anatomical subjects, by Mr. MAGENDIE, are inserted in the last Number of his Physiological Journal.† In the one, which he has illustrated with two plates, Mr. Magendie gives a description of the organs destined to tighten or relax the membrane of the tympanum and the ossiculi of the auditory apparatus, in man and mammiferous animals; and, in the second paper, he has detailed the anatomical appearances of a dog, born with one eye only, and without a mouth. One of the singular facts connected with the latter examination is the total absence of the optic nerve from the eye, notwithstanding which the retina, which has hitherto been considered as the expansion of that nerve, was found *in situ*, and perfectly formed.

On the subject of the organ of hearing, we must not omit to mention the work of Professor WEBER, which, though published some months ago, has only lately reached this country.‡ We shall give a short analysis of the first part of it at the earliest opportunity.

Mr. CHARLES BELL has communicated to the Royal Society of London his inquiries on the anatomy and physiology of the facial nerves, in which he has endeavoured to unravel their complicated arrangement, and prove the utility of their numerous plexusses.

The elementary work on anatomy, by Mr. SHAW, for the particular use of students,§ has already been noticed in the

* BECLARD, *Additions générales à l'Anatomie de Bichat.* 1821.

† *Sur les Organs qui tendent ou relachent la Membrane du Tympan, et la Chaine des Osselets de l'Ouïe, dans l'Homme et les Mammiferes.—Anatomie d'un Chien Cyclope et Astome.* (Jour. de Physiologie, No. 4, p. 341,—374.

‡ *De Aure et Auditu Hominis et Animalium.* Part I. *De Aure Animalium aquatilium.* Auctore E. H. WEBERO. Cum tabulis œneis. Lipsiæ.

§ *A Manual for the Student of Anatomy; containing Rules for displaying the Structure of the Body, so as to exhibit the elementary Views of Anatomy, and their Application to Pathology and Surgery.* By JOHN SHAW. London, 1821.

Medical and Physical Journal. We then ventured to predict that all those pupils who throng to the metropolitan mart of anatomical knowledge, would not fail to provide themselves with a book which had been purposely written for them, and which offered to them so many peculiar advantages: we, in fact, gave it as our opinion that Mr. Shaw's book would supersede every other book *of the same kind.* A critic, who writes in a contemporary journal, smiled at our prediction, and, with a degree of conscious superiority, which may be natural in a writer of a quarterly series, expressed his conviction "that the book would, on the contrary, be more popular among those classes for whom it was least designed,—namely, those at a distance from the metropolis, who are slowly initiating themselves, at county hospitals and infirmaries, in the knowledge of anatomy and dissection." How far this critic will prove correct when a second edition shall have appeared, we will not take upon ourselves to state; but the ink that recorded his opinion could scarcely have been dry when the passing event of the day, respecting the sale of the first edition of Mr. Shaw's book, proved how fallacious that opinion was; for, in the course of a few days, upwards of two hundred copies, constituting the greater part of the edition, were distributed among the eager pupils of the metropolis! This fact we have from the best authority, that of the author himself.

There is a very curious memoir, in the Number of the Journal Complementaire for August,* by a Mr. Gerdy, on the organization of the heart, which deserves consideration. The subjects treated at full length in this paper are—I. The albugineous tissue of the heart; II. The carneous tissue of the same organ. The former, according to Mr. Gerby, consists of—1, auricular zones; 2, the tendons of the valvular columns; 3, arterial zones; 4, the fringed margins of the aortic and pulmonary artery at their origin; 5, the tendons of the sigmoid valves of those arteries. The latter, or the carneous tissue, is different in the ventricles and in the auricles. The author describes them both very minutely, and then proceeds to the enumeration of several other very interesting subjects connected with the structure of the heart.

A case of a man having two distinct noses has been mentioned by Dr. Bidault de Villiers, who twice examined the individual presenting that deformity, at different times, very particularly, and has clearly described the phenomenon in the tenth volume of the Journal Complementaire.†

In the ordinary mode of performing the operation of litho-

---

* *Memoire sur l'Organization du Cœur.* Par S. N. Gerdy, élève Naturaliste du Gouvernement.

† September 1821, p. 183.

tomy, or in puncturing the bladder *per perineum*, it is of the most essential importance to be well acquainted with the origin, connexion, and extent, of the membranous partition between the pelvis and the perineum. The medical public in America has of late been much interested in several discussions that have taken place respecting the anatomy of those parts; and we have, from time to time, announced in this Journal the result of those discussion. Dr. STEVENS, of New-York, and Mr. WILLIAM ANDERSON, surgeon in Nova-Scotia, have recently communicated the result of several dissections, intended to settle the question in point.* The latter gentleman has also published a separate account of his examination of the male pelvis.†

A new division of the animal tissues in the human body has been proposed by Professor MAYER, of the Prussian university of the Lower Rhine, under the name of *Histologia*. The professor distinguishes—1, the lamellated tissue, to which he assigns ten distinctive characters; 2, the cellular fibrous tissue; 3, the fibrous tissue; 4, the cartilaginous system; 5, the osseous system; 6, the glandular tissue; 7, the muscular fibre; and 8, the nervous tissue.‡

The Croonian lecture, read since the Easter recess of the Royal Society, by Sir EVERARD HOME, presents some new microscopical observations respecting the structure of the human eye, and the use of the marsupium in birds, which will be received with pleasure by the medical public.§

New and accurate editions of MORGAGNI's celebrated work on Pathological Anatomy are in course of publication both in Paris and at Milan. In the latter city, Dr. FARRERI has undertaken a new edition of the anatomical work of Mascagni. The Italian translation of Dr. BAILLIE's work on Morbid Anatomy, by ZANINI; and the copious description given by LUIGI FANZAGO, keeper of the museum of the university at Padova, of several pathological preparations in that collection, show that the Italians do not neglect the study of this interesting branch of anatomy.‖

Some remarkable aberrations in the distribution of blood-vessels, in addition to those already recorded by Professor MECKEL, have been noticed by that distinguished physiologist, in the body of a male child, who died aged nine months.¶

---

* *On the Fascia Iliaca.* By A. H. STEVENS, M.D.—(New-York Medical Rep. Vol. 6, No. 11.)

† *A Description of the Anatomy of the Urinary Bladder, with its Appendages, concerned in the Operation of Lithotomy.* By W. ANDERSON.—(Philad. Journ. No. 3.)

‡ *Bibliotheque Germanique,* No. 8, tom. 2.

§ Not yet printed.

‖ *Memoria sopra alcuni Pezzi Morbozi conservati nel Gabinetto Patologico dell' I. R. Università di Padova.*

¶ *Sur quelques Aberrations remarquables dans la Distribution des Vaisseaux.* Par J. F. MECKEL—(Jour. Comp. p. 32, Nov. 1821.)

Professor Mojon, of Genoa, has published some anatomico-physiological observations on the epidermis.*

A singular case of phimosis in an infant has been described by Dr. Gerard, of Beauvais. The prepuce was found to adhere around the corona glandis, and likewise at the meatus urinarius. The space between these two extremities of the glans was wholly free from adhesion.†

In the same periodical publication will be found an account of the removal, from the inner surface of the rectum, of a pediculated fungous tumor, that had been the source of great suffering to the patient, a lady, and which was very distinct from an hemorrhoidal tumor. The extirpation was practised by means of a ligature. Dr. Laracine, of Paris, was the medical attendant on the occasion.

The morbid changes which take place in the human body in consequence of disease, and the presence of which during life is not always suspected, form an interesting subject of inquiry. On this account, the readers will peruse with satisfaction Prof. Hensinger's *Memoire sur les Monstruosités de la Rate;* and the Dissertation on the Hypertrophia of the Liver, by Victor Murat, contained in the Bulletins of the Medical Society of Emulation. The case of carnification of the gall-bladder in consequence of the presence of a large calculus, recorded by the Editor of this Journal, it is hoped, will not be deemed altogether destitute of interest.‡ Nor is the case of distension of the œsophagus, found after death by Mr. Purton, devoid of instruction.§ We may also mention in this place the case of ossification of the ventricular aorta, so well described by Mr. Howell, in the October Number of our Journal.

Under the head of monstruosity may be mentioned the instance of a double fetus, recorded in this Journal for November, the preparation of which is in the possession of Mr. James Barton, Dale-end, Birmingham; as well as the example of a child being born at the Maternité, in Paris, with the whole surface of the body wrinkled like that of a very old man. In a memoir, remarkable for its simplicity, Mr. Bourgeois has detailed some curious facts respecting the urachus;‖ and Dr. Duparcque, of Paris, has, in a similar manner, given the description of an external serous cyst, situated at the inferior extremity of the trunk in a new-born infant, forming a large

---

* *Osservazioni Notomico-fisiologiche sull' Epidermide.* Di B. Mojon.

† *Observation sur un Phimosis extraordinaire.* Par M. A. Gaspard, Medecin à Beauvais.—(Bulletins de la Société d'Emulation, August 1821.)

‡ *London Medical and Physical Journal,* October 1821.

§ *An extraordinary Case of Distension of the Œsophagus.* By F. Purton, F.L.S. —(London Med. and Phys. Journ. vol. xlvi. p. 540.)

‖ *Recherches Anatomiques sur l'Ouraque.* Par M. Bourgeois.—(Journ. Gen. de Med. Oct. 1821.)

tumor, which impeded birth until after its laceration, and the consequent evacuation of the fluid it contained. The tumor was of a nature distinct from that known under the name of spina bifida.* No example of the latter disease on record can equal that which we mentioned in the last Number of our Journal, and the subject of which is still living under the care of Mr. JUKES, of Westminster.

In Comparative Anatomy we have but few facts to record. Dr. DAVY's paper on the Urinary Organs of two Species of Rana, contained in the last volume of the Philosophical Transactions, deserves an honourable mention in this place. We may also refer to the Description of the Nerves which go to the Mustachios of the Phoca, given by Mr. ANDRAL, jun. Nor is the paper of Sir EVERARD HOME to be passed over in silence, which details the peculiarities that distinguish the manatee, or dugong, of the West from that of the East Indies.

In one of the recent Numbers of the New-York Medical Repository, we find it announced that a species of acipenser, or sturgeon, has been discovered on the coast of New-York, the vertebral column of which exhibits the membrano-gelatinous structure, except as regards the lateral and spinous processes, all of which are tipped with bone. This curious organization is accompanied with another peculiarity: the vertebræ, though neither bony nor cartilaginous, show a disposition to separate into joints.

### II. PHYSIOLOGY.

The next important object to that of acquiring a precise knowledge of the human fabric, is to endeavour to ascertain in what manner, and by what laws, each part of its machinery is made to act in a state of both health and disease, and at different periods of life. This is precisely the object of physiology, the study of which has been most eagerly and successfully pursued in modern times.

In looking over the hundred periodical publications and other works which lie scattered before us, we can scarcely determine on what new physiological fact to dilate first, among those that have been made known during the last six months.

Dr. DEWAR, of Edinburgh, in a very ingenious essay, has endeavoured to show that the laws of chemistry exert an influence on *living* operations; and that, therefore, in studying the phenomena of physiology, we should not lose sight of the relation of living action to chemical laws.†

---

* *Observation sur un Kyste Sereus externe chez un nouveau né, &c.* Par Mr. DUPARCQUE.—(Biblioth. Medical. Sep. 1821.)

† *The Influence of Chemical Laws on the Phenomena of Physiology.* By H. DEWAR, M.D. &c.—(Edinburgh Med. Journal, October 1821.)

Dr. Roulin has given us the result of some theoretical and experimental inquiries into the mechanism of the movements and attitudes of man. His memoir contains several curious facts and luminous inductions, and may be considered as an attempt to introduce, once more, the use of mathematics into the study of physiology. The author has prefaced his inquiries with a very clear, though succinct, historical account of the opinions and labours of the iatromathematical school, of which Borelli was the most distinguished member.* Dr. Roulin has also written on the mechanism of muscular ruptures; respecting which he observes, that a muscle may be ruptured by any force tending to separate the two points to which its extremities are attached, and also by the overpowering action of an antagonist muscle.†

Mr. Down, in a paper inserted in this Journal, has published some observations tending to show how far life can be identified with heat. He combats the opinions of Mr. Hunter, and questions the soundness of the inductions of Mr. Brodie from his experiments on this subject. Mr. Down, however, seems not to have comprehended the views of either of those physiologists.

The Transactions of the Royal Society, which, of late, have been rich in physiological papers, again offer, on the present occasion, several memoirs of the same nature.‡ The first of those contained in the volume for 1821, is on the Black Rete Mucosum of the Negro, by Sir E. Home. The second relates to two singular facts concerning the physiology of Generation, by the Earl of Morton and Dr. Giles; and the third (being the Croonian lecture,) embraces several microscopical observations on the brain and nerves, on the valves discovered in the branches of the vas breve, and on the structure of the spleen, by Sir Everard Home and Mr. Bauer. Dr. Davy has supplied the fourth paper, in which he has given a physio-anatomical account of the Urinary Organs of two species of the genus rana. All these papers have been noticed by us in some of the latest of our preceding Numbers.

Mr. Magendie has inserted, in his Journal of Physiology, a memoir on vomiting, by Mr. Piedagnel, resident hospital-pupil of the first class in Paris; in which the author endeavours to combat the objections started by Mr. Bourdon§ against the theory of vomiting, settled by the experiments of Magendie,

* *Recherches, theoriques et experimentales, sur le Mechanisme des Mouvemens et des Attitudes de l'Homme.* Par F. Roulin, M.D.—(Jour. de Physiol. Exper. No. 3.)

† *Du Mecanisme des Ruptures Musculaires.* Par F. Roulin, M.D.—(Journal de Physiol. Exper. No. 3.)

‡ *Philosophical Transactions of the Royal Society of London, for* 1821. Part I.

§ See *London Medical and Physical Journal*, vol. 45, p. xvi.

who has contended that the stomach takes no active part in that operation.

The effects of herbaceous vegetable aliments on the stomach of man have been observed by Dr. GASPARD, during the calamitous famine which desolated several districts in France, in the year 1817. The herbaceous vegetables used on that occasion were wild-sorrel, nettles, succory, thistles, the tops of French beans, and the sprigs of young trees. These herbs were minced, and boiled in the same manner as spinnage; but, when they became too hard, the inhabitants expressed a thick juice from them, by straining them through a piece of linen. The uniform result of such a regimen, continued for a long time, was a general serous diathesis or universal anasarca; and, in six cases, which proved fatal, dissection showed that the stomach and intestines were extremely contracted.

On physiological subjects, the Medico-Chirurgical Transactions of the last half-year have only presented us with a single paper, by Mr. SWAN, on the Transmission of Sounds to the Organ of Hearing by impressions made on the facial nerves.

The experiment made by Dr. COPLAND, of Walworth, upon himself, with a view to ascertain the effects of oleum terebinthinæ on the animal economy, is not more remarkable for its novelty, than for the display of sound and extensive erudition which it has drawn forth from the author. Dr. Copland's paper will be found one of the best that have been written in this country for a long time on physiological subjects; and approaches, nearer than any we have lately read, to the numerous memoirs of equal merit which our transmarine neighbours have of late years been in the habit of reading before the Institute, and other highly respectable societies, of France.*

The effect of belladonna thrown up the rectum, in the form of an enema, in producing delirium, or rather somnambulism, has been well described by Dr. SARLANDIERE.† Human rumination is of so rare occurrence, that the additional case mentioned by Mr. NESSE HILL will be read with interest.‡ The fact noticed by Baron PERCY, on more than one occasion, of wounds emitting a phosphorescent light, which in some cases last for several days, is one which physiologists will find some difficulty in explaining.§ The recent observations of Dr. HEINRICH, of Ratisbonne, on phosphorescence in general, should be consulted in this case; and, no doubt, much information will be derived from their perusal.‖

---

* See *London Med. and Phys. Journal*, July 1821, p. 107.

† *Revue Medicale*, and *London Med. and Phys. Journal*, July 1821.

‡ *London Med. and Phys. Journal*, No. 269, p. 119.

§ *Travaux de l'Academie des Sciences*, par le Baron CUVIER.

‖ *Bibliotheque Universelle*, xv. p. 247.

The memoir of Mr. Chevreul on the Influence which Water exerts on the Physical Properties of various solid Animal Substances, will be found to contain several hints likely to be of service to physiologists, in investigating various phenomena occurring in the living body ;* and the galvanic experiments recently made at Glasgow on the body of a criminal, will confirm many of the inductions that have been drawn from similar experimental investigations on two or three former occasions.†

Perhaps one of the most singular papers that have appeared during the last six months on this branch of medical knowledge, is that of Mr. Gerdy, whom we have had occasion to mention in the preceding section, in which he has analyzed the various phenomena of life. We have as yet seen only the first part of this paper ; but, to judge from the methodical manner in which he has noticed, described, and arranged the phenomena in question, we expect to derive much pleasure from the perusal of the remainder.‡

A subject which has often perplexed the ingenuity of physiologists, is that of the various sympathies acting on the organs of the human economy. So many are the morbid phenomena dependent on these sympathies, that the practical physician has not unfrequently been baffled in his expectations and judgment respecting diseases under his treatment. One of the principal difficulties inherent to this question, is the ascertaining of the positive existence of what has been called sympathy, as an active agent, in any of the phenomena of life, whether morbid or otherwise, under consideration. The difficulty, in fact, consists in being acquainted with the semiology of sympathies. This very difficulty is precisely what Dr. Gelcen has endeavoured to remove, by his observations contained in the eleventh volume of the Journal Complementaire ; to which we request the attention of our readers.§ Mr. Caffin, a naturalist, at Orleans, has treated the same subject in a recent paper inserted in the Journal général de Medicine, for September 1821.

The notion that mercurial remedies pass from the stomach and from the skin into the circulation, and penetrate, equally, the solids of our animal economy, has been so prevalent, that Dr. Rhades, of Halle, must find it a matter of no little difficulty to support a contrary opinion. This physiologist has undertaken to repeat the experiments, once made by some of the Berlin professors, with a view to ascertain whether mercury, rubbed on the surface of the body of animals, passed into their blood ; and, like them, he concludes, that neither mechanical

---

* *London Med. and Phys. Journal*, December 1821. † *Op. cit.*

‡ *Essai d'Analyse des Phenoménes de la Vie.* Par M. Gerdy, Aide d'Anatomie à la Faculté de Medecine, &c.—(Journal Comp. October 1821.)

§ *Journal Complementaire*, tom. 11. p. 2.

agents nor chemical tests can detect the presence of the metal under the circumstances in question.*

In another part of the present section of our Essay, we mentioned that Dr. Dewar considered the various physiological phenomena to depend on the action of chemical laws. Dr. HARLAN, of Philadelphia, on the contrary, has written a paper purposely to shew the inefficacy of those theories which are founded upon the doctrines of chemical life. His inquiries are limited to the consideration of the generation of animal heat, which, he has endeavoured to show, has no connexion with the function of respiration. He smiles at the hasty conclusion of Dr. WILSON PHILIP, that heat is a secretion, and, like all secretions, the effect of the nervous influence on the blood. This hypothesis, Dr. Harlan properly observes, could only have been framed on limited views of the animal economy.†

In the department of experimental physiology, we must not omit to allude to the physiological remarks of Prof. KRIMER on various subjects:—1, on the Secretion of Urine; 4, on the Strength of Muscles; 3, on the Effect of Nervous Lesions in disturbing the animal Functions; 4, on the Blood, and its colouring Principle; 5, on the Relation between the Form and Organization of the labyrinthic Nerve, (portio mollis of the seventh pair,) and its Functions.‡

The numerous, and some of them very original, experiments made and published by Dr. SCHROEDER, of Groningen, on the Coagulation of Blood, should likewise be adverted to in this part of our Essay, as tending to throw a very considerable light on the nature of that fluid in different constitutions, at different periods of life, and under various circumstances of health and disease. The experiments were varied in every possible way, and lead to some curious conclusions applicable to physiological medicine.§

### III. ELEMENTARY AND PRACTICAL MEDICINE.

The elements of medicine ought to consist in the observation of facts, and the derivation of proper inductions from them collectively, on which we may ground those principles that are to guide us in our practical career. This method of studying medicine is equidistant from the metaphysical abstractions of university education, where the ideology of the art only is taught; and from empiricism, which notes every passing event,

* *Experimenta circa Quæstionem an Hydrargyrum externe applicatum in Corpore et presertim in sanguine reperiatur.* Halle. 4to. 1821.

† HARLAN *on the Generation of Animal Heat.*—(Philadelphia Journal, August 1821.)

‡ *Gazette Medico-Chirurgicale d'Ehrhart.*

§ *Dissertatio Physiologico-Medica sistens Sanguinis Coagulantis Historiam cum Experimentis ad eam illustrandam institutis.* Groningæ, 1821.

and draws from *each* isolated fact conclusions for *general* application.

Elementary works on medicine are now rarely to be met with. The spirit of research has taken a turn for individuality. Generalizations are no longer in fashion. Mr. Broussais, whose name we have so often repeated, has endeavoured, in a work which we have analyzed, to overthrow every existing system of nosology; and, had he been better qualified for the office of reformer he has assumed, there is no doubt but that he would have succeeded in nullifying the various prejudices which have hitherto impeded the progress of medicine.*

Tommasini and Mantovani, in Italy, in their respective works on Inflammatory Fever, have also powerfully co-operated in giving a more decisive character to the practice of physic.† Sir Gilbert Blane had, at the same time, pointed out the errors to be avoided in the pursuit of medical inquiries; and our readers must have seen with pleasure, from the account we have given of the second edition of his Medical Logic, that the elements of right discrimination are to be found within us, and need only to be properly used in order to make good our progress towards the attainment of truth.‡

The subjects which have more particularly engaged the attention of physicians within the last six months, are those of pestilential fever and cholera morbus. Numerous are the works that have appeared on each of those destructive disorders; and it would be a difficult task to enumerate, at the same time, the many pamphlets, essays, and papers, published on the same subject. The former disease, occurring in countries nearer home, than those in which the cholera morbus rages, has necessarily engrossed most of the attention, not only of the medical practitioners, but of the different governments of Europe. Spain has long been smarting under the dire effects of that pestilential scourge; and, whether the fever which has lately prevailed in Catalonia, and is still actively existent in Andalusia, be or be not contagious, it is nevertheless true that, in its nature and effects, it approaches nearer to a pestilential distemper than to a common disorder spreading epidemically.

Dr. Robert Jackson has published his remarks on the Epidemic Yellow-Fever which has appeared, at intervals, on the south coasts of Spain since the year 1801.§ This gentleman relates that the importation of the disease, though still credited by the authorities and mass of people, has never been proved

* *Examen des Doctrines Medicales, &c.* 2 vols. 2d edit. Paris, 1821.—See also *London Med. and Phys. Journal* for September 1821.

† *Considerazioni Patologiche sull' Infiammazione es ulla Febbre continua,* del Dr. Tommasini.—*Lezioni sulle Infiammazioni,* del Cav. P. Mantovani. 3 vols.

‡ *London Med. and Phys. Journal,* September 1821
*Medico-Chirurgical Review* No. 7, p. 485.

by evidence; and he concludes, from a few facts which he has himself collected, and which he desires us to believe, that the contagion of yellow-fever does not attach itself to clothing. On the whole, however, there is a pretty general admission, throughout the work, both in words and sentiments, of the Andalusian fever being contagious. Yet Dr. JAMES JOHNSON, who is better acquainted than any other person with Dr. Jackson's work, and who ought to have learned from its perusal what, we understand, his experience has not given him an opportunity of learning,—namely, that instances of persons who have lived in apparent good health in simple epidemic atmospheres, and who have become sick soon after their association with affected individuals, are both numerous and well-marked,—has unaccountably volunteered his services on the side of the non-contagionists, the plurality of whom speak on the subject without any personal experience; just as Dr. Johnson himself has done, who has never seen the yellow-fever, upon the inocuousness of which he has descanted.* We, who formed part of a commission charged with the examination of the yellow-fever at Malaga, in 1804, and remained in that town the best part of 1805; who cruised in the West-Indies during the prevalence of the same epidemy among the islands in 1809 and 1810, where we were affected by the disorder; and visited Cadiz when the same fever raged in that city, at a subsequent period; are not inclined to speak so *positively* as to the non-importation, and the non-contagious nature, of the disease in question. Our two last Numbers are full of documents, of the most irrefragable authority, against the doctrine of those writers on whom we have been commenting; and, if Dr. Jackson, who was certainly not at Barcelona while publishing his book in London, is to be believed, Dr. Johnson *suadente*,—it will be difficult for the latter gentleman to disbelieve the evidence of Dr. PARISET, whose generous and noble devotion placed him in a situation to form a much more decided opinion on the reality of both mediate and intermediate contagion in yellow-fever.†

Dr. Johnson's letter, on the subject in question, has been successfully answered by several writers in the public papers, and by an article inserted in the Number of our Journal for December.

Mr. O'HALLORAN, who followed Dr. Jackson in his expedition to Cadiz, and who, we understand, is since gone to Barcelona, though at a too-late period to study the disease, has also published a small work on the Yellow-Fever of Anda-

---

* *London Med. and Phys. Journal,* November 1821.

† See Dr. PARISET's Letter, from Barcelona, in the *London Med. and Physical Journal* for December; and in the French *Moniteur* for November.

lusia; of which we may at some future period give a succinct review.*

Baron LARREY has also taken the field as a writer on Yellow-Fever; thus increasing the number of French authors who have lately absorbed the whole attention of the faculty on the continent with their lucubrations on that subject.

Nor have the Americans remained behind-hand on this interesting topic; and their discussions, or controversial disputations, are at least as numerous, if not as violent, as those of the European physicians. The Philadelphia Journal and the New-York Repository are, indeed, full of them; but we shall only mention a work by another Dr. JACKSON, the president of the Board of Health at Philadelphia, who has described the yellow or malignant fever which occurred in that city in 1820.†

The Spanish physicians have also written copiously on the subject of the fever which devastates the richest portions of their country. The work by HURTADO DE MENDOZA is by far the most important; and not the less remarkable for the assertions which it contains in favour of the non-contagionists.‡

Coupled with the subject of yellow-fever, we may bring forward that of the epidemic fevers that are known to prevail, occasionally, in various districts of our islands. We are not aware, however, that the last six months have given birth to any work on those fevers worthy of being commented upon in this place.§

In France, a Neapolitan physician has shown, with the assistance of appropriate quotations and much erudition, that the doctrine which Broussais endeavours to promulgate is taken, almost verbatim, from BAGLIVI, the illustrious Italian physician, who flourished at the commencement of the last century, and practised in Rome.‖ The assigning as a cause of fever a topical affection, particularly of the stomach, is so strongly marked in Baglivi's writings, that we are almost tempted to consider Dr. Broussais as a mere plagiarist. To those who have read the summary account which we have given of that gentleman's doctrine, it will be needless to point out the almost *literal* similarity which exists between his promulgated principles and those of Baglivi, contained in the following quotation.

---

* *A brief View of the Yellow-Fever, as it appeared in Andalusia during the Epidemic of* 1820.

† *An Account of the Yellow or Malignant Fever, as it occurred in the City of Philadelphia in* 1820. By S. JACKSON, M.D. 1821.

‡ *Nueva Monografia de la Calentara Amarilla, o Tratado Medico Teorico pratico sobre la Verdadera Naturalezza, &c. &c.* Por Don M. HURTADO DE MENDOZA.—*Memoria sobre la Fiebre Amarilla contagiosaque a regnado en Espagna.* Madrid, 1821. Anonymous.

§ *Edinburgh Med. Journal,* No. 69, p. 623.

‖ *Histoire de quelques Doctrines Medicales comparées à celles du Dr. Broussais.* Par M. FODERA. Paris, 1821.

"Pluries vidi, dari febres nonnullas in ipso principio graves statim, statim periculosas, et periculosis etiam stipatas symptomatibus. Has vulgo vocat malignas, idest à venenifero productas humore: cujus imaginarii et commentitii veneni de causa, innumera statim præscribunt spirituosa, aromatica, alexipharmaca, calida, volatilia, et mille id genus inter se pugnantia remedia; quibus quod præcavere volunt periculum advocant et augent.

"Ipse enim, ut vera fatear, quæ diligenti observationi et matura didici meditatione, a duabus potissimum causis, malignas has febres pendere observavi INFLAMMATIONE VISCERUM, et ab apparatu pravorum, crudorumque humorum in primis viis, &c."

And, farther on, when speaking of the paroxysm of fever, Baglivi describes its symptoms, one would imagine he was reading the tenets of the modern *Ecole Physio-Pathologique*. "Statim apparere videbis linguæ ariditatem, pulsus exiguos, extremorum frigus, anxietates et alia id genus, quæ *malignam febrim* (*ataxique* of Pinel,) denotarent; sed revera non *sunt malignitatis effectus*, (Broussais attacks precisely in the same language the author of the Nosographie Philosophique,) verum enim stomachi ab exaltato humore *irritati*, lacessiti, et afflicti; qua cessante irritatione, et stomachi indignatione compositâ, præfata cessant accidentia."*

Even where Broussais piques himself on absolute and indivisible originality,—namely, in having strongly inculcated the necessity of avoiding strong purgatives in visceral inflammation, and of relying on bland laxatives, diluted acids, and enemas, we find him copying the very words of the Italian physician we have just been quoting, who first descanted on the same points, with equal force. "Fuge nimium remediorum copiam, (exclaims Baglivi;) fuge purgantia tanquam pestem; si tamen ingens fuerit pravorum humorum in primis viis apparatus, qui morbum producat et foveat; fomentis et clysteribus minuendus; et si hæc non fuerint satis, potio aliqua tamarindata leniens danda, dummodo tamen gravis ardor, et extremæ inflammationis rabies, contrarium non requirant. . . . . In febribus ardentibus sal prunellæ specificum est. . . . . Aliquando utor nitro perlato, interdum vero antimoniato cum hordei decoctione."

Poor Dr. Broussais has enough to do to parry all these heavy blows. They come thick upon him, from all quarters. Dr. ANTHENAC, of Montpellier, has addressed several letters to him† on the subject of his attack against the French physicians, which it will be no easy task for him to answer.

---

* BAGLIVI, *Praxeos Medicæ*, lib. i. *De Febribus Malignis et Mesentericis.*

† *Defence des Medecins Français contre le Docteur Broussais, auteur de la Nouvelle Doctrine, &c.* 1re. livraison. Paris and Montpelier, 1821.

Two English translations of important practical works in medicine, possessing considerable merit, should be noticed as belonging to the series of books published during the period we are considering. The one is by Dr. GOOCH, and relates to the Acute Hydrocephalus of Children;* the other by Dr. FORBES, purporting to be a condensed translation of Laennec's classical work on Diseases of the Chest.† We shall speak of both at greater length on some future occasion.

We would also fain say something of two other works, which have been lying before us for review for a considerable time; but we must reserve our opinion for another opportunity. We trust we shall do justice to both. Contemporary journalists, having no pretensions to any *personal* acquaintance with the subject of which those works treat, have reviewed them already, and passed judgment upon them. How far the authors themselves are satisfied with that judgment, we pretend not to divine: but we trust that they will not feel sorry to have fallen into the hands of other critics, who, having lived in pestilential countries, and among the pestilent, must, necessarily, be somewhat better judges of their performances.‡

We have already noticed at full length Dr. REID's book on Hypochondriasis, and Dr. REEDER's on Diseases of the Heart, to need entering, here, into a repetition of their contents.§

From Padova, we have received a continuation of those excellent Clinical Reports which were first begun by the Caval. BRERA, and have since been continued by several of his assistants. Nothing tend more to advance practical medicine than these statements of cures and failures, observed during a given period, among patients who are under the special treatment of professors of an university, for the exclusive instruction of pupils about to enter into practice.‖ The example is worthy of imitation in this country, where numerous hospitals and other medical charities afford opportunities for observations and the trial of new medicines, equalled no where on the continent. We shall endeavour to encourage, as far as simple individuals can do so, a practice from which the utmost benefit must be derived. The Report which we published on a former occa-

* *A Treatise on the Hydrocephalus Acutus, or Inflammatory Watery Head.* By L. A. GOLIS; translated from the German, by W. GOOCH, M.D.

† *A Treatise on the Diseases of the Chest, in which they are described according to their Anatomical Character, &c.* Translated from the French of R. T. H. LAENNEC, M.D. with Notes, by J. FORBES, M.D. Physician to the Penzance Dispensary.

‡ *Researches into the Laws and Phenomena of Pestilence, &c.* By THOMAS HANCOCK, M.D. 1 vol. 8vo. pp. 379. 1821. *The History of Plague, as it has lately appeared in the Islands of Malta, Gozo, Corfu, &c.* By J. D. TULLY, Esq. Surgeon to the Forces, 1 vol. 1821.

§ See *London Med. and Phys. Journal*, December 1821.

‖ *Prospetto de Risultamenti osservati nella Clinica Medica della R. I. Università di Padova, nel corso dell' anno scolastico* 1820.

sion* shall be resumed, and continued yearly in this Journal; and we trust to be able to add to it, from time to time, Reports also from a physician to a general Dispensary of great practice, and from the medical officers of the most extensive charity for the treatment of diseases of children in this country.

The French physicians have of late directed much of their attention to the treatment of intermittent fevers by means of a new remedy, obtained from the bark of cinchona, combined with sulphuric acid. We allude to the sulphate of quinine; respecting the superiority of which over common bark in those diseases, we have seen numerous public attestations. This fact seems to afford the *rationale* of the effects said to be produced by a mixture of cinchona and sulphuric acid, which English practitioners are occasionally in the habit of prescribing.† Mr. MORSON, chemist, of Fleet-market, has detailed an easy method of obtaining the sulphate of quinine, in the last Number of the Monthly Repository.

In an Essay of so limited extent as the present is intended to be, we cannot be expected to enter, in detail, into all the various communications bearing on the theory and practice of medicine, which are to be found in periodical publications, both foreign and domestic. The majority of these communications, indeed, deserve but a moderate share of attention; but we cannot omit calling the attention of the readers to Sir GILBERT BLANE's proposal for treating chronic hydrocephalus by means of pressure made with bandages, properly applied to the head; to Dr. MACLEOD's observations on the property which the prussic acid seems to have of exciting salivation, and ulceration of the buccal mucous membrane,—observations, the justice of which we have since had opportunities of verifying; to Mr. BAMPFIELD's remarks on the superiority of small over large doses of colchicum, in the successful treatment of the gouty paroxysms: all which papers will be found in the Medical and Physical Journal for the last quarter. Nor can we mention, with less satisfaction, Dr. KNOX's case of pericarditis, and his observations on the adhesion of serous membranes;‡ and the successful treatment of erysipelas by venesection, recorded by Dr. DUNCAN, junior, of Edinburgh.§

That phlegmasia will occasionally pervade the tunics of blood-vessels, is a fact for the knowledge of which we are entirely indebted to modern practitioners. Mr. SMERDON has related a case of arteritis terminating fatally;* another example

* *A Report of the Practice of Midwifery at the Westminster General Dispensary in* 1818.—(See London Med. and Phys. Journal, vol. xliv. p. 231.

† See Dr. BALLY, Dr. DOUBLE, Dr. CHOMEL, Mess. DUVAL and DUFOUR, *Considérations pratiques et Observations sur l'Emploi du Sulfate de Quinine dans les Fièvres intermittentes;* in the Revue Medicale, for July, September, and October, 1821.

‡ *Edinburgh Med. and Surg. Journal,* No. 69, p. 516. § *Id.* p. 537.

of inflammation of the arteries is recorded by Dr. JOHNSON;* and a third case, with many appropriate remarks, is to be found in one of the late French Journals, by Dr. BAYLE.†

The attention of the profession being once called to the specific inflammation of the mucous membranes, we shall in time be able to collect many valuable facts on that subject, particularly with regard to those of the stomach and intestines. A case of acute gastritis is inserted by Dr. DESLANDES, in the Bibliotheque Medicale for September: it terminated in gangrene and death in less than sixty hours. The author of the present Essay has likewise detailed an instance of well-marked gastro-enteritis, which ended fatally in less than four days; and cases of ulceration of the stomach are recorded by Dr. CHEESEMAN, in the last American Journals; and by Mr. DESGRANGES, of Lyons, in the Journal Général. The case of an illustrious deceased, in whose stomach the same affection was observed, is too well known to need being repeated in this place.

### IV. CHIRURGERY.

We shall be brief in what we have to say respecting this branch of the healing art.

One of the most important improvements in surgery that have been brought forward during the last six months, is, undoubtedly, the new mode, proposed and executed by Sir ASTLEY COOPER, for extracting small calculi from the urinary bladder, without the employment of cutting instruments. A case, in which eighty-four such calculi were thus removed by Sir Astley from the bladder of a patient, was read before the Medico-Chirurgical Society of London.‡ The instrument employed is a sort of forceps, which, when introduced into the bladder like a common catheter, opens itself into two blades, kept apart by a stilette until one or more of the calculi are grasped within them; when, on the stilette being withdrawn, the calculus remains confined, and may be drawn through the urethra.

On the subject of lithotomy, we must not omit to mention the classical and interesting paper by Mr. MARTINEAU, of Norwich;§ nor the case, though unsuccessful, related by Mr. TRIPE, in a communication to us, in which a calculus of great magnitude, occupying the whole of the cavity of the vescica, was extracted. Had Mr. Tripe known the ingenious contrivance of Mr. Earle for breaking large stones in the bladder,

---

* *London Med. and Phys. Journal*, Dec. 1821.
† *Bibliotheque Medicale*, September 1821.
‡ *London Med. and Phys. Journal*, November 1821.
§ *Med. Chirurg. Transactions*, vol. xi. *On Lithotomy.* By P. M. MARTINEAU, Esq. &c.

he would have been less perplexed how to dispose of that which presented itself to him during the operation.*

But the individual to whom the thanks of the profession are principally due, in reference to improvement in the mode of cutting for the stone, is, without question, Professor VACCA, of Pisa, whose candid and modest statement of six successful cases of lithotomy, practised, with some advantageous alterations, according to the suggestion of Mr. SANSON, of Paris, of penetrating into the bladder by the rectum, we have had the satisfaction of laying before our readers.† It will be recollected that, when Mr. Sanson first proposed his new mode of lithotomy, the thing was deemed impracticable, and that, after two unsuccessful attempts only, on the part of Dupuytren, it was rejected by that eminent surgeon, and subsequently forgotten. The Italian surgeons, Barbantini and Vaccà, however, have shown, by their perseverance and success, that we should never despise that which, in our hands, does not immediately succeed. Mr. WHEELER has communicated to the Editor of the Quarterly Journal of Foreign Medicine, a relation of some cases of lithotomy which occurred in the hospitals at Paris, chiefly for the purpose of remarking on the best means of suppressing the hemorrhage sometimes attendant on the operation.‡

Another example of a partial amputation of the foot, so contrived as to preserve the shape and usefulness of the limb, has been added by Mr. DUNN to those originally brought forward by Mr. HAY, of Leeds, and Mr. A. C. HUTCHISON. Mr. Dunn removed part of the tarsus and metatarsus in his case.§

While physicians, by means of repeated trials, are endeavouring to establish the claims of iodine to the cure of bronchocele, surgeons proceed in the removal of that deformity according to Professor QUADRI's method of treating it with the seton. A curious paper, containing much information relative to this subject, and a successful case operated upon in the Quadrian manner by Mr. A. C. HUTCHISON, will be read with great satisfaction.‖

The checking of the stream of circulation in the carotid by ligature, in cases of aneurism of that artery, is now performed with as little hesitation as any other surgical operation of more frequent occurrence. Although terminating in death, the case related by Mr. COATES, in which he tied the carotid artery affected by an aneurismal tumor of the largest kind, will be perused and referred to with interest and advantage.¶

---

* *London Medical and Phys. Journal,* October 1821, p. 171.
† *Idem.* September 1821.
‡ *Quarterly Journal of Foreign Medicine,* No. XI. for July.
§ *Medico-Chirurgical Transactions,* vol. xi. p. 11.
‖ *London Med. and Phys. Journ.* Nov. 1821; and *Med.-Chir. Trans.* vol. xi. p. 11.
¶ *Medico-Chirurgical Transactions,* vol. xi. p. 11.

Some interesting remarks on Necrosis, by Mr. A. C. Hutchison, will be found in the October Number of the Medical and Physical Journal; and a case in which a portion of bone, six inches in length, affected with that disease, came away from the middle of the tibia of a young man of scrofulous habit, has been related in an Italian periodical publication. The case terminated favourably.*

The employment of ustion in certain diseases is becoming very frequent on the continent. Cases in which this powerful therapeutic measure has been resorted to with success, are to be met with frequently in the German and French journals. Mr. Priou, of Nantes, and Dr. Desgranges, of Lyons, have added to the evidence already before us on this point. A case of cerebral affection, one of articular rheumatism, a third of epigastralgia, a fourth of submammary pain with dyspnœa, another of obstinate cephalalgia, and a foul ulcer, have all been conducted to a salutary termination, by these practitioners, by the application of fire. Mr. Priou has entered into some instructive inquiries regarding the history and various modes of practising this surgical operation.†

Several papers on nosocomial gangrene have been published, both here and abroad, within the last half-year, and some new remedies proposed to avert its formidable effects. Tracheotomy has been performed successfully in three cases. In the one, by Dr. Klein, of Stuttgardt, for the removal of a bean; in the other, by a gentleman, whose name we could not catch when the paper was read at the Medico-Chirurgical Society, in which a pebble, of the size and figure of a bean, was extracted from the trachea. In both cases, fresh evidence has been brought forward of the comparative insensibility of the trachea *proper*, when contrasted with the exquisite irritability of the larynx. In a third case, related by Mr. Porter, the operation was successfully resorted to for the cure of cynanche laryngea.‡

Sir A. Cooper has successfully extirpated a large adipose tumor, situated midway between the umbilicus and ensiform cartilage. When removed, it measured one yard and a quarter in circumference, and eighteen inches around its neck. It weighed about thirty-seven pounds ten ounces.§

An instance of laceration of the colon, supposed to have been produced by the simple efforts of defecation, and followed by peritonitis and subsequent death, has occurred to Mr. Fievee, of Paris.||

---

* *Memoria Patologico-Clinica sulla Necrosi e sopra un raro osseo Processo.*—(Annali Universali di Medicina, 1821.)

† *Journal Général de Médecine*, vol. 76, p. 37.

‡ *Medico-Chirurgical Transactions*, vol. xi. Part 2. § *Idem.*

|| *Journal Général de Médecine*, vol. 76, p. 203.

There is a singular relation in one of the latest French journals, of a foreign body, looking like an *os calciné*, having been extracted from the articulation of the knee, through an appropriate incision. The author would have found, had he read the admirable treatise of Mr. BRODIE on Diseases of the Joints, that what he has considered as an *os calciné*, and consequently as a foreign body, was perhaps nothing else than a *disguised* loose cartilage.* There is more evidence of surgical tact and skill in this gentleman's relation of an operation for an aneurism of the anterior branch of the temporal artery, which proved successful.†

Of the works strictly surgical published within the last half-year, we have to report, on the whole, very favourably; and, foremost amongst them all, duty, justice, and friendship, bid us mention the productions of the late lamented Mr. WILSON. The public knew, but did not appreciate, the great worth of that man. Justice has not been done to him, neither by those who were personally strangers to him, nor by a few among those who had the pleasure of his acquaintance. What avails it strewing faded roses on the tomb of him whom we have slighted during life? Mr. Wilson's classical work on the Urinary Organs will be referred to by *honest* and grateful pupils, for successive years, as the best model of compilation, in which simplicity and candour are to be found, instead of the turgid pomposity, or the affected Tacitus-like aridity of expression, of some of his contemporaries, who cry out "*Inveni!*" when reciting the neglected discoveries of their predecessors.

In France, Mr. BOYER has added a seventh volume to his treatise on Surgical Diseases, equal in value and importance to the former volumes of that interesting publication. It is consecrated to the consideration and exposition of the chirurgical maladies of the neck, thorax, and, partly, of those of the abdomen.‡

Professor RICHERAND has given us a new edition of his Surgical Nosography, which is greatly enriched by new matter, particularly respecting those new and bold operations which many of the English and French surgeons have successfully performed in diseases hitherto deemed incurable.§

Mr. JAMES's Observations on the Nature and Treatment of Inflammation, and Mr. SWAN's Dissertation on the Treatment

---

* *Observation d'un Corps étranger dans l'Articulation du Genou.* By S. T. GASTE, D.M.—(Journal Univ. Nov. November 1821.)

† *Journal Universel des Sciences Medicales*, October 1821.

‡ *Traité des Maladies Chirurgicales et des Operations, qui leur conviennent*, tom. vii. Paris, 1821.

§ *Nosographie Therapeutique & Chirurgicale.* Par le Chevalier RICHERAND. Nov. edit. 1821.

of Morbid Local Affections of the Nerves, have each been reviewed by us within the last half-year.

Diseases of both eyes and ears have had, within the same period, their historiographers. We need not mention TRAVERS and VETCH, as authors of valuable works on ophthalmic surgery; and we shall take an early opportunity of giving a detailed account of a considerable work, by Dr. ITARD, of Paris, on the Morbid Affections of the Auditory Organs.*

Nor should the very recent performance of Baron LARREY, on various points of surgery, be forgotten. This veteran and indefatigable army surgeon has positively no equal, either on this or on the other side of the Channel in his peculiar department.†

### V. OBSTETRICAL MEDICINE AND SURGERY.

Though certainly not the least important of the medical sciences, obstetricism has not, of late, received that attention, in this country, which it fully deserves and loudly calls for. Our own Journal, in this respect, has, like every other English medical journal, somewhat neglected the subject; and we are determined, from a conscious conviction of its importance, to devote a considerable portion of our future labours to the advancement of Midwifery. In the literature of this department there has been little to notice for the last six months. In France, one or two works on the Practice of Midwifery have appeared. The first is by an eminent midwife, Madame LA CHAPELLE, head matron at the hospital of la Maternité in Paris, whose death we regret to have to announce at the same time.‡ The second is written by Dr. MOULIN, and consists of four large synoptical tables, in which every possible case is recorded that is likely to occur in the practice of midwifery; and the corresponding rule or plan of treatment for accomplishing the delivery is severally noted.§

In this country, we are not aware that any specific work on Midwifery has been published in the course of the last half-year. Dr. ONSLOW continues to insert his curious and useful researches into the history of Obstetricism, particularly as they are connected with the progressive improvement of that art in England, in the Monthly Medical Repository.‖

---

* *Traité des Maladies de l'Oreille et de l'Audition.* Par Mr. ITARD. 2 vols. Paris, 1821.

† *Recueil des Memoires de Chirurgie.* Par le Baron LARREY. 1 vol. 8vo. 1821.

‡ *Pratique des Accouchemens, ou Memoires et Observations choisies sur les points les plus importans de l'Art.* Par Madame LA CHAPELLE, Sage Femme en chef de la Maison d'Accouchement de Paris. 1 vol. 8vo. Published by Dr. DUGES, her nephew.

§ *Cours Pratique d'Accouchemens, avec une nouvelle Nomenclature des Presentations et Positions du Fetus, designée sous le Nom generique de* Pelvi Fœtale. Par E. MOULIN, Docteur en Medecine. Paris, 1821.

‖ *Obstetrical Researches.* By M. ONSLOW, M.D.

In a pamphlet of some few pages, Dr. POWER has announced several new views on certain points of the female economy, relative to menstruation, and a new species, as the author calls it, "of abortion not heretofore described." A very accurate and just idea of the pamphlet may be formed by perusing either of the reviews that have appeared in the journals of the day. The *new species* of miscarriage described by Dr. Power is attributed, by him, to a want of a proper proportion of oxygen in the blood of the mother, and is to be prevented by inhaling a certain quantity of that gas medically administered. To this *new species* of miscarriage "delicate females in HIGH LIFE" are said to be peculiarly liable; and Dr. Power, in support of his novel and luminous theory, has brought forward TWO cases of a Mrs. A. and a Mrs. N., who, having repeatedly miscarried before, miscarried no longer when they inhaled a certain quantity of his vital air.*

Practical facts in midwifery have been somewhat more frequently detailed in the public journals, and in other publications. The most extraordinary one of this kind is the case recorded by Dr. M'KEEVER, of ruptured vagina during parturition, so as to admit the escape of a yard and a half of the intestinal tube; which, after passing through the gangrenous process, came away without destroying the life of the patient, who has since been afflicted with an artificial anus in the vagina. The child had been delivered with the crotchet, the operation lasting two hours!† We recollect being present, about two years ago, at the dissection of a poor woman, who died immediately after being delivered with the forceps, in whom an extensive laceration (it was called spontaneous rupture,) of the vagina was found between the bladder and uterus, which had evidently destroyed the patient's life.

Mr. SCOTT, of Norwich, a very sensible and expert practitioner in midwifery, has related the case of a woman, who, during a severe labour, lost a circular portion of the uterus, which was exhibited at one of the meetings of the Medico-Chirurgical Society. On examination, *per vaginam*, about three weeks after delivery, Mr. Scott found a continuous cavity, without any distinction between the vagina and uterus. The patient recovered.‡

On the subject of ruptures of the uterus, Mr. PURTON has communicated a paper to the Editor of this Journal, in which he relates the case of a woman who died undelivered of her

---

* *Essays on the Female Economy.* By JOHN POWER, M.D. See also *London Medical and Physical Journal*, Sept. 1821.

† Dr. M'KEEVER's *Case of Ruptured Vagina*, in the 3d volume of the Dublin Transactions of the King's and Queen's College of Physicians.

‡ *Medico-Chirurgical Transactions*, vol. xi. Part 2.

tenth child, in consequence of a rupture of the uterus, through which the fetus escaped into the cavity of the abdomen. Mr. Purton was not allowed to have recourse to the Cæsarean operation, either before or after death.* A case has been related by Mr. BAILEY, where the whole of the placenta was expelled before the child. Mr. Bailey, very properly, and without any loss of time, delivered the woman by turning the child.† A fresh instance of what has been called spontaneous evolution of the fetus is mentioned, in the same journal, as having occurred in the Infirmary of the parish of St. George's.

A distinguished French surgeon, Mr. BECLARD, performed the Cæsarean operation in July last, at Paris; an account of which was read at one of the meetings of the Royal Academy of Medicine. The incision was made in the direction of the linea alba. Both the mother and child did well.‡

The displacement of the womb during pregnancy is one of the most distressing occurrences to which a woman is liable at that period. Mr. W. BARTLETT, of Great Bedwin, Wiltshire, has given the particulars of a case of Retroversio Uteri, in the London Medical Repository for July. The accident is minutely and accurately described, as well as the treatment employed, which consisted in introducing the hand into the vagina, and a finger *in ano*, so as to replace the womb in its natural situation, by means of a gentle pressure. This method was first recommended by Mr. GREGOIRE. It has been stated, by a Dr. OLINET, that the umbilical cord may be so disposed around the fetus as to increase its volume, and thereby impede natural parturition. The Doctor has narrated an instance of the kind, which had fallen under his notice, and where it became necessary to afford assistance by means of the forceps.§ A much more serious obstacle to the completion of labour, is the unnatural and, it may be called, spasmodic contraction of the womb around the neck of the fetus, after the head has passed through the os tincæ. Professor HALL, of Baltimore, has reported an extraordinary case of difficult labour, from the cause just mentioned. Dr. BATTZELL, the consulting physician on that occasion, in endeavouring to pass his fingers between the uterus and the body of the child, for the purpose of turning, found the womb so forcibly drawn around the neck of the fetus, that delivery by the application of the forceps became impracticable, and the woman died undelivered. ¶ We were called in

---

* *London Med. and Phys. Journal*, November 1821.

† *London Medical Repository*, December 1821.

‡ *Nouveau Journal de Médecine*, August and September, 1821. *Seances de l'Academie Royale de Médecine.*

§ *Journal Univer. des Sciences Med.* 1821.

¶ *Medical and Chirurgical Review*, No. 6. p. 439.

consultation, towards the close of last summer, by a gentleman in extensive practice, in the neighbourhood of Russel-street, in a case precisely similar. The Assalini forceps, made longer than that surgeon employs, was applied, and a firm grasp obtained; but traction produced no change in the relative position of the child; and, on examining with the left hand, the womb was found to impede the descent of the fetus, by its firm contraction around the basis of the neck. Embriotomy was eventually had recourse to, notwithstanding the high situation of the head, and the patient was delivered without much difficulty. Here the labour had lasted upwards of sixty hours before instruments were had recourse to, and the liquor amnii had been discharged the preceding day.

Few instances of posthumous parturition have ever been recorded. Dr. Schenk, counsellor to the King of Prussia, and physician at Siegen, attended a lady who died of chronic bronchitis, in the first week of the ninth month of her pregnancy. On the arrival of her medical attendants, who were summoned from some distance to her, they found that the patient had been dead about three hours, and that no movement of the child could be discovered through the abdominal parietes. The Cæsarean operation was instantly proposed, but peremptorily rejected by the friends. The body of the deceased was left a whole day, undisturbed, on the bed: on the following day the clothes were changed, preparatory to her being buried. Nothing remarkable was observed on that occasion; but when, on the third day, the body was uncovered for the purpose of its being placed in the coffin, a dead male child was observed lying between the legs of the deceased; the placenta hanging partially out of the vagina. Dr. Schenk very properly regrets that, when the Cæsarean operation was objected to, he did not proceed to ascertain how far it would have been possible to deliver the woman in the usual manner.*

There is no complaint so troublesome, or so frequent, though in itself not dangerous, as the milk-abscess in the breasts of lying-in women. It occasions both suffering and inconvenience, and will occasionally be found to continue for months, in spite of the utmost skill on the part of the medical attendant. Mr. Burne, of Edinburgh, aware of the truth of these observations, has directed his attention to the subject; and we venture to say, that our readers will derive much information in the perusal of his paper on the best mode of obviating and treating the disease in question, and of forming a good nipple.†

---

* *Journal Complémentaire*, August 1821.

† *Observations on Mastodynia, or Milk Abscess.* By John Burne, President of the Royal Society of Edinburgh.—See also the *London Med. and Phys. Journal* for August 1821.

In that branch of obstetrical medicine which relates to female complaints and the diseases of children, we have the satisfaction of noticing the publication of the second part of Mr. CLARKE's Observations on Utero-vaginal Diseases attended by Discharges.* We shall not lose time in laying an extended account of this book before our readers.

Dr. DESRUELLES, of Paris, in a memoir of considerable merit, has fully detailed the difficulties which present themselves in forming a correct diagnosis of the diseases of children. To remove these, Dr. Desruelles proposes to consider the age of childhood as divided into four periods, each of which, he contends, is liable to peculiar disorders. These disorders are again subdivided according to temperament and peculiarities of constitution; and thus, by narrowing the field of examination, the practitioner (Dr. Desruelles thinks,) will arrive more speedily at a knowledge of the disease.†

The treatise on the Whooping-cough, *coqueluche*, (not croup, as stated by a contemporary,) of the late Dr. MARCUS, has been translated into the French language. The object of the author is to prove, by reasoning, as well as by necroscopical inspections, that the whooping-cough and bronchitis are one and the same disease. The author contends that pertussis is always inflammatory, in which he is decidedly wrong: whereas, in bronchitis, inflammation is always present. The identity of the two complaints, therefore, is not to be sustained.

The physical education of children is now so much more attended to than in former times, that any new publication on that subject is sure of being well received. Dr. RATIER has published a memoir on the Physical Education of Children, which has obtained the premium proposed by the Royal Society of Medicine of Bourdeaux. His considerations on the various points of his inquiry are arranged in chapters, and the subjects treated in them are the following: 1, air; 2, dress; 3, bathing; 4, frictions; 5, food and drink; 6, medicines; 7, excretions; 8, exercise; 9, sleep; 10, passions.

We shall conclude this section with an honourable mention of a small, but valuable, tract, by Mr. PLUMBE, on a disease peculiarly incidental to children, and by some considered the opprobrium of medical-surgery: we allude to the porrigo capit s, and its varieties. As the work will shortly fall under our more immediate consideration, we shall content ourselves, on the present occasion, with observing, that it reflects credit on the author.‡

* *Observations on those Diseases of Females which are attended by Discharges.* By CHARLES MANSFIELD CLARKE. Part II. London, 1821.

† *Journal Universel des Sciences Medicales*, September 1821.

‡ *A Practical Essay on Ring-worm of the Scalp, Scalled Head, and Porrigo.* By S. PLUMBE, Member of the Royal College of Surgeons. 1821.

### VI. HYGIENE, JURIDICAL MEDICINE, AND MEDICAL POLICE.

The first paper of any interest which occurs to us on the art of preserving health, in the period we have embraced, is one by the learned President of the Horticultural Society, giving an account of a new mode of maintaining an equable and salutary temperature in the air of rooms for consumptive patients.* To this may be added the memoir of Mr. SYLVESTER, on the best method of warming and ventilating houses.†

Under the head of Hygiene we are disposed to place the highly-important subject of vaccination. The last Report from the National Vaccine Department in London to the Home Secretary is, in many respects, satisfactory ; but we contend, that too many concessions, and some of those too hastily made, are to be found in that document, of failures, which should never be recorded unless properly authenticated.‡ It is, indeed, a singular circumstance, that the sound of alarm should alone have been raised in the country which gave birth to the most brilliant discovery that ever graced the annals of inventive genius. But granting, even for one moment, (as we have stated in another place,) that all the failures that have either been whispered about, or loudly proclaimed, were true to their whole extent, what are they when distributed among the millions of cases of permanent preservation from that scourge, the small-pox, which have been recorded in every part of the civilized globe, since JENNER first drew the prophylactic lymph from the ruminating quadruped? Does not every other human contrivance, the best imagined, the most zealously supported, present a greater number of anomalies, that defy prospective calculation ? Have not the annals of inoculated small-pox, when most in vogue, offered double, nay, more than double, the number of instances where the artificial disease proved an ineffectual barrier to the irruption of the more formidable reality ? Let us, then, be wise, and make good use of our blessings. If we look to foreign nations, where is the kingdom, the province, the town, the village, in which a single note of complaint against the effectual virtue of vaccine has been heard ? The Central Committee for propagating Vaccination in France have told us, in their last Report,§ that the multiplied experiments and numerous observations, made during more than twenty years, had proved, in the most positive manner, that vaccination was most efficacious in preventing the small-pox, and might, possibly, become the means of completely eradicating that disease. The committee

* *London Medical and Physical Journal,* July 1821.
† *The Quarterly Journal,* No. xxii.
‡ *London Medical and Physical Journal,* August 1821. § *Id.* November 1821.

had also heard of pretended cases of failure; but, instead of yielding, readily, their belief to them, they directed proper inquiries to be made into their reality; and the majority of those cases disappeared like visions at noon-day.

Some valuable and highly-interesting documents have lately been printed, but not published, by the prefect of the department of the Seine,* from which it is our intention to select many important facts for insertion in our Journal, at an early opportunity. In the formation and arrangement of works like these, the French are indeed unrivalled. Dr. LACHAIRE has also published some reflections on the population of Paris, from an examination of the progress of which, he thinks, that many precious facts may be derived, not altogether destitute of interest for the philosopher who is engaged in inquiries of public hygiene.†

A volume, containing many curious details respecting the history and practice of medical gymnastics, has recently appeared in Paris, from the pen of Dr. LONDE; of which the periodical journals, and the reporters at the Royal Academy of Medicine, speak in flattering terms of commendation. The author has considered the subject under two views,—1, as relating to hygiene; 2, in relation to therapeutics.‡ The various exercises are first described; their influence on the animal economy, and on health, is explained; and rules are given by the author to regulate their uses according to the constitution and strength of the individual, their age, sex, habit, and temperament.

An account has been given, in one of the French journals, of the experiments, or rather of the attempt, made by ROSENFELDT, (an illiterate German enthusiast, with the whole of whose proceedings we happen to be acquainted,) to inoculate himself with the plague, in the Greek hospital at Constantinople, towards the close of the year 1816. The account is written by Mr. PEZZONI, an eye-witness, who, with ourselves and others, was present at similar experiments made by the late Dr. VALLI. Rosenfeldt pretended to possess a preservative against the contagion of the plague, and died a miserable victim of his credulity.§

A general Report, for the preceding year, from the Committee of Public Health in Paris, (*salubritê publique*,) is lying before us; and we are happy to remark the great zeal with

---

* *Recherches Statistiques sur la Ville de Paris, et le Department de la Seine.* 1 vol. 8vo. Tables.

† *Reflexions Medico-Philosophiques sur le Mouvement de la Population de Paris.* Par le Docteur LECHAIRE.—(Journal Comp. Oct. 1821.)

‡ *Gymnastique Medicale, ou l'Exercise appliqué aux Organes de l'Homme, d'après les Lois de la Physiologie, de l'Hygiene, et de la Therapeutique.* Par CH. LONDE, M.D. Paris, 1821. 1 vol. 8vo. pp. 351.

§ *Journal Complémentaire,* August 1821.

which the members have taken up the subject of charlatanry. By great perseverance, they succeeded in convicting some of the most refractory and most impudent quacks before the Courts; and have gone far in checking the encouragement which empiricism never fails to meet amongst the unwary and the ignorant, by *preventing the publication of advertisements respecting quack medicines, and promoting, as much as possible, the knowledge of every trial and condemnation of quacks and impostors,* among the lower classes.*

In Juridical Medicine, and the branches connected with it, the principal works are those of ORFILA, of which an account has already been given in this Journal;† and of Dr. SMITH, respecting which we shall have to speak more at length hereafter.‡ Mr. BRIAND, of Paris, has also published a Manual of Juridical Medicine, containing extracts from the most esteemed works on that subject, such as those of Mahon, Foderé, Dr. Marc, &c. The ingenious classification of wounds by the latter has been adopted by Mr. Briand, as the surest guide in cases of surgical injuries coming before the tribunals. Another work of great merit is that of Mr. CAPURON, our much-esteemed instructor on juridical medicine relative to the obstetrical art. We may hereafter enter more fully into the merits of this production.

A case of poisoning by arsenic, successfully treated, has been related in the last two Numbers of our Journal, by Mr. HUME, the able chemist, of Long-acre; and an instance has been reported by us of a person, who, having drank, by mistake, a quantity of bleaching liquid, died with all the symptoms of having been poisoned.§

Although published in February, the new experiments of ORFILA, on corrosive sublimate, bleaching liquid, delphinum, opium, and nux vomica, became known in this country only within the last six months. The author has considered each of these subjects in reference to juridical medicine, particularly with regard to the difficulty of ascertaining the presence of those poisonous substances, after death, when mixed in the stomach with other liquids or solid food.‖

The question of reform and abuses in the medical profession has again been agitated in the different journals in this country; but we are not aware that any new light has been thrown on

---

* *Rapport général sur les Travaux du Conseil de Salubrité, pendant l'année* 1820. Paris, 1821. And *Journal général*, September 1821.

† *Leçons faisant Partie du Cours de Medecine legale.* Par M. ORFILA. Paris, 1821.

‡ *The Principles of Forensic Medicine, systematically arranged and applied to British Practice.* By S. G. SMITH, M.D. 1 vol. 8vo. 1821.

§ *London Med. and Phys. Journal*, for November 1821.

‖ *Nouveau Journal de Medecine, &c.* Also *Medical Intellig.* July 1821.

the subject, or that a safe and sure mode of correcting the revolting abuses at present existing has been proposed.*

The detection of the various frauds practised by the venders of articles of food, is one of the first duties of medical police. Professor DAVY, of Cork, has invented an ingenious instrument, by which the frauds committed in the sale of skimmed milk may be easily detected.†

### VII. MEDEOGRAPHY.

We have adopted this name, as more expressive and more consonant with the formation of every other technical name in medicine, than that of *materia medica*, in lieu of which we wish it to stand. In fact, why should we retain the anomaly of a Latin noun and adjective among the denominations of different branches of medical science, which have themselves been designated by appellations of exotic origin, indeed, but so adapted as to suit the genius of the English language? What reason can be urged for retaining the name of materia medica, instead of Medeography, after anatomy, surgery, pathology, physiology, botany, chemistry, &c.? Under this head, the history and virtues of medicines are exclusively considered, whether in regard to their preparations, mode and time of exhibition, indication, and doses; or with respect to the particular action that they are known to exert, or are supposed to have, on the animal economy.

Four important questions in medeography have been discussed, among many others of minor interest, during the last half-year, in this country and on the continent. 1. The real action of hydrocyanic acid in certain diseases; 2, the effects of colchicum in diseases of excitement, and the superiority of small over larger doses of that medicine in the cure of the gouty paroxysm; 3, the virtue of cubebs in the treatment of gonorrhœa; 4, the influence of iodine on glandular and scrofulous disorders.

Each of these points has been the subject of much controversy and of many contradictory statements. While Dr. MAGENDIE, in France, Professor BRERA, in Italy, Dr. BEHR and Professor HELMEKIN, in Germany, Dr. GRANVILLE, in England, and Dr. NANCREDE, in America, openly avow their conviction of the salutary effects of the prussic acid in pectoral complaints, founded upon several years' experience, and supported by numerous cases; Dr. ELLIOTSON, Dr. MACLEOD, and a notable reviewer, who confesses that he has never tried that

* See Mr. YEATMAN and AN OLD PRACTITIONER in the *London Med. and Phys. Journal*;—IGNATUS, and the Correspondence of the Apothecaries' Hall, in the *Repository*.

† *Philosophical Magazine*, for November 1821.

medicine, deny it any visible action on those diseases. Dr. MACLEOD's statement is a candid one, and, as far as it goes, conclusive of the fact that, in several cases of senile and chronic cough, the medicine in question failed to produce relief. This gentleman has also observed that the hydrocyanic acid has the property of exciting salivation; a fact worthy of consideration, and which has been confirmed by the observations of the author of the present Essay.*

On the subject of colchicum, Mr. BAMPFIELD, one of the surgeons of the Royal Metropolitan Infirmary for Sick Children, has written a paper in support of the practice of administering small doses of colchicum, and of the specific virtues of that medicine in subduing the paroxysm of gout;† which has drawn from Dr. SCUDAMORE a reply, in defence of his particular doctrine and mode of administration of that medicine.‡

The property which cubebs are supposed to possess in speedily curing gonorrhœa, has been variously debated; but the subject is entirely confined to the profession in this country. The evidence, on the whole, seems favourable to the medicine.

Not so, however, with regard to the use of iodine, first proposed by Dr. COINDET, of Geneva, and since used, pretty extensively, in Germany, France, and England. Dr. Coindet will scarcely admit any case of failure; while others of his fellow practitioners and countrymen not only proclaim such failures, but complain of its deleterious effects, so as to induce some of the canton governments to forbid its use, except under certain restrictions. Dr. DEL CARRO, of Vienna, on the contrary, says that neither failures nor accidents of any kind have happened, to his knowledge, in about one hundred cases which have been treated with iodine; while, in France, opinions are yet balanced as to the real properties of that substance on the human frame.§ In this country, no decisive result on this subject has been obtained; or, if it has, it has not been publicly stated. Sir Andrew HALLIDAY, domestic physician to the Duke of Clarence, has indeed announced that, in three cases of scrofulous tumors, in which he prescribed the tincture of iodine, that medicine proved successful;‖ and an anonymous correspondent, in the same Journal, has borne testimony to the efficacy of that remedy, though not to the extent of Sir Andrew; but we have had an opportunity of hearing the details of an experiment, made by two physicians of great reputation and extensive practice, which go to prove iodine to be an inert medicine in the complaints for which it has been recommended. In this

* *London Med. and Phys. Journal*, September 1821.
† *Op. cit.* November 1821. ‡ *Op. cit.* December 1821.
§ *Revue Medicale*, September 1821.
‖ *Med. Repository*, September 1821.

conflict of opinions, a singular circumstance is to be remarked, namely, that a substance, admitted, on all hands, to possess high powers of excitement, should be recommended in such a class of disorders as *scrofula*. Are not scrofulous diseases produced, and are they not always accompanied, more or less, by inflammatory action? In conclusion, we ought not to lose sight, that the violent effects of iodine on the animal economy rest on the authority of men of high character; and that Dr. NORDHOF, of Aubone,* and Dr. GOLIS, of Vienna,† urge the profession to be very cautious in the administration of that substance.

The use of oil of croton, in constipation of the bowels, has also been a subject of attention during the preceding six months. It is known that a single drop of this oil produces copious, and sometimes violent, purging. This medicine, however, is by no means new. MURRAY, in his Apparatus Medicaminum, has given the botanical description of the plant from the seeds of which the oil is obtained; and RUMPHIUS has detailed, pretty extensively, the virtues of both the grain and the oil.

A fresh instance has been brought forward, by Mr. THOMSON, of Whitchurch, to prove that we are not always correct in blindly following the recorded maxims of old, respecting the proper doses of medicines to be administered. This gentleman has related a case of paraplegia, attended by anasarca, in which the latter symptom was quickly removed by means of *four-and-twenty*-grain doses of gamboge at a time.‡

The readers of the London Medical and Physical Journal will have perused, with satisfaction, Dr. COPLAND's paper on the employment of terebinthinous remedies in various diseases, inserted in one of our late Numbers. The same subject has been treated by an American author, in one of the recent periodical publications of that country.§

Mr. BRETON recommends the bark of the pomegranate tree in cases of tænia; and has descanted on the efficacy of the bark of the swietenia febrifuga, as a substitute for that of cinchona.‖

Since the discovery of quinine and cinchonine, and their respective salts, many have been the reports made on the efficacy of those substances in the treatment of pernicious and intermittent fevers. Dr. MAGENDIE bears his own testimony to the virtue of the sulphate of quinine;¶ and Dr. CHOMEL, Dr.

---

* *Allgemeinen Medic. Annalen*, 1821.
† *Medicinische Chirurg. Zeitung*, 1821.
‡ *Medical Repository*, November 1821.
§ *New-York Repository*, vol. vi. No. 4.
‖ *Medical and Chirurgical Transactions*, Vol. xi. Part ii.
¶ *Fièvre intermittente pernicieuses guerie paur une seule dose de Sulfate de Quinine.* Par M. MAGENDIE.—(Jour. de Physiologie, No. 4.)

DOUBLE, and many others, speak in unqualified terms of approbation of those remedies.*

Being desirous to set at rest the question respecting the real effects of acetate of lead on the animal economy, Mr. GASPARD has made several experiments, from which it results that the medicine in question is a most dangerous poison, slow and insidious; that it exerts a peculiar inflammatory action on the bowels, and, in some degree, even on the lungs.†

The subject of tar fumigation in diseases of the aërial passages has again been brought forward by Mr. WARD, with a report of successful cases.‡

The tincture of common poppy has been proposed as a substitute for laudanum, by Dr. WILSON, of the United States of America.§ Dr. MEAD, of the same country, has published an Experimental Inquiry into the Chemical Properties and Medicinal Virtues of the Spirœa Tormentosa of Linnæus.||

From the results of Mr. SERULLA's experiments on the preparations of antimony used in medicine,¶ it would appear that a small proportion of arsenic is invariably present in them. If this be a fact, it behoves the different faculties of medicine, here and elsewhere, to direct that other methods should be employed for obtaining the preparations in question than those which are at present in use; and which, it would appear, are liable to such fearful inconveniences.

### VIII. CHEMISTRY AND PHARMACY.

Neither of these sciences has produced, during the last half-year, results worthy of minute or attentive consideration. The curious inquiry into the laws of permutation which electricity and magnetism reciprocally follow, first announced by Professor OERSTED, of Copenhagen, continues to engage the pen of many distinguished men of science, both here and abroad. We have, in another department of the present Number, inserted a short account of the progress of this new science (electro-magnetism), taken from Mr. BRANDE's Journal; through whom we have been favoured with the necessary wood-cuts to illustrate that paper.** But, by far the best view of the subject is that contained in the Annals of Philosophy, in which the original discovery of electro-magnetic forces by the Danish professor is distinctly described, and every successive step, through which that inquiry has gone, is accurately and clearly traced.††

---

* *Nouveau Journal de Médecine*, and *Journal général de Médecine.*

† *Journal de Physiologie*, No. 3.

‡ *London Med. and Phys. Journal*, November 1821.

§ *Philadelphia Medical Journal*, August 1821.

|| *New-York Medical Repository*, vol. vi. No. 3.

¶ *Journal de Pharmacie*, September 1821.

** See COLLECTANEA MEDICA, Art. 2.

†† *Annals of Philosophy*, September, October, and November, 1821.

Mr. I. G. CHILDREN has communicated to the public his analysis of the pigment with which the hieroglyphic figures on the celebrated sarcophagus from the tomb of PSAMMIS, now in the British Museum, are filled. The colouring matter of that pigment is oxide of copper.* An improvement in Woulfe's apparatus has been suggested by the Marquess RIDOLFI, which, in some degree, does away the necessity of using distinct tubes of safety, during the operation of distillation with that series of vessels.† Both the French and the Americans have endeavoured to support a claim of priority in the application of steam-engines to navigation; but a fact has been ascertained by, and is to be forthwith published in a work of, Mr. PARTINGTON, of the London Institution, which sets the disputed point at rest. From a patent, the specification of which is placed at the beginning of a rare tract, in the British Museum, it appears that JONATHAN HALLS expressly stated, in 1736, that he should apply the steam-engine for the conveyance of vessels. The work in question is illustrated by an engraved plate, representing a steam-boat towing a large vessel; and is accompanied by an account of the apparatus employed for that purpose.‡

Mr. BERARD has been engaged in a course of experiments to determine what chemical changes take place during the maturation, ripening, and decay, of fruits of various kinds.§ Some novel calculations on the extent to which the divisibility of matter may be carried, under particular circumstances connected with chemical solution, have been proposed by an anonymous writer in the Quarterly Journal of Science.‖ Mr. I. F. DANIELL has invented a new pyrometer, which promises, from its utility in measuring the higher degrees of heat, to be of eminent service to chemists and manufacturers.¶ A new Dictionary of Chemistry has been published by Dr. URE, of Glasgow, which has deservedly received the commendation of many of our contemporaries.** We wish, also, to recommend to the attention of the junior students in chemistry a work of much merit, (quaintly entitled One Thousand Experiments on Chemistry,) which contains more valuable and practical facts, in speculative matters, the arts, and the manufactures of this country, as well as copious illustrations of the principal theorems of chemical science, than are to be met with in many recent

---

* *Annals of Philosophy,* November 1821.

† *Giornaladi Fisica di Brugnatelli.* 1821.

‡ *Annals of Philosophy,* December 1821.

§ *Annales de Chimie et de Physique.* 1821.

‖ No. xxii.

¶ *On a new Pyrometer.* By J. F. DANIELL, F.R.S.—(Quarterly Journal, No. xxii.)

** *A Dictionary of Chemistry, on the basis of Mr. Nicholson's.* By ANDREW URE. London, 1821.

chemical works of superior pretensions.* In the United States, Dr. GORHAM has also published an elementary work on chemistry, entitled the Elements of Chemical Science, in two volumes; of which, public report speaks favourably. A new edition has appeared of THENARD's Traité de Chimie, in four volumes : this is by far the most classical book of the kind. Dr. MACULLOCH has written a paper on the method of obtaining potash from the stalks of potatoes, which is full of curious details, and refutes the exaggerated statements put forth by the French chemists, in 1817, on this subject.† The new compounds of sulphur and cyanogene, mentioned by BERZELIUS;‡—the combinations of subsulphate of alumine and potash, examined by RIFFAULT;§—the analysis of verdigris, by Mr. PHILLIPS;||—the formation of alcohol by fluoboric gas, by Mr. DESFOSSES, of Besançon;¶—the new combination of oxides with chlorine, iodine, and cyanogene, by Mr. GRANVILLE;**—the experiments of Mr. FARRADAY on the new compounds of chlorine and carbon, and on a new compound of iodine, carbon, and hydrogen;—are a few of the many subjects of interest in chemical science, that have been debated during the last half-year. To these may be added the classical paper of Dr. HENRY, of Manchester, on olefiant gas; in which there are many stubborn facts and experiments, militating against some of the propositions advanced by Mr. BRANDE, who had previously treated the same subject.†† The paper of Mr. JOHN DALTON, on the analysis of mineral waters, deserves also the utmost consideration.‡‡

By far the most curious and important paper in chemistry, to be found in the foreign journals of the last half-year, is that of Mr. SERULLAS, on the alloys of potassium, and the existence of arsenic in the antimonial preparation used in medicine. We shall only mention one fact contained in the memoir in question, relative to the decomposition of tartar emetic by the action of fire. Mr. Serullas, by exposing that triple salt in a covered crucible to a red heat, has obtained an alloy of potassium and antimony, which burns vividly when exposed to the air, pyrophorus-like, and becomes oxidized. We are surprised that this paper has not yet attracted the notice of some of our scientific contemporaries.§§

---

* *One Thousand Experiments in Chemistry and the useful Arts.* By COLIN MACKENZIE. 1 vol. 8vo. 1821.

† *Journal of Sciences, No.* xxii.

‡ *Annales de Chimie,* No. xvi. p. 23.

|| *Annals of Philosophy.*

¶ *Annales de Chimie,* No. xvi. p. 72.

** *Op. cit.*

†† *Philosophical Transactions,* 1821.

‡‡ *Manchester Memoirs,* vol. iv. New series.

§§ *Journal de Pharmacie,* September 1821.

Scientific and analytical Pharmacy has been much neglected of late years, in England. In France, Germany, and Italy, on the contrary, (but particularly in the two former countries,) the subject continues to be most zealously attended to; and the results are such, that numberless vegetables have been analyzed, new medical preparations proposed, and a spirit of research manifested, which does infinite credit to the individuals interested in those investigations. It is sufficient to name Pelletier, Caventon, Henry, Robiquet, Boullay, Braconnot, Berard, Cadet Gassicourt, Virey, and Vogel, all of them *pharmaciens*, to be satisfied that, in analytical pharmacy, there are fewer persons of equal claims to public consideration.

In another part of the present Number, we have translated, at full length, a paper of Messrs. FENEULLE and CAPRON, giving an analysis of the black hellebore.* The *helleborus hyemalis* had already been analyzed by VAUQUELIN. And, in the London Medical and Physical Journal for November, will be found an account of the active principle obtained by Mr. ANDRAL, jun. from the white hellebore, to which principle he has given the name of *veratrine*.

The separation of the active principles from the different species of cinchona bark, by Messrs. PELLETIER and CAVENTOU, and the preparation of the different salts resulting from the combination of those bases with acids, form an epoch in analytical pharmacy. We have detailed these discoveries, as they were published in France, in several Numbers of our Journal. The analysis of pepper, by Mr. Pelletier, is also deserving of consideration.† From the pen of Mr. HENRY, the principal director of the general laboratory of the Parisian hospitals, we read, with pleasure, a memoir on the colouring matter of saffron; in which, besides the process of separating that principle from the plant, the author has detailed its specific properties and a mode of obtaining the volatile oil contained in the saffron. The application of the facts brought forward by Mr. Henry, on this occasion, to the preparation of different tinctures in which saffron is present, must be obvious; particularly with regard to what is called the compound wine of opium, or the laudanum of Sydenham.‡ In the Bulletin de la Société d'Emulation for September last, Mr. DESFOSSES has announced the presence of a narcotic principle in the *solanum nigrum*; an analysis of which he has given, with the process for obtaining the principle itself, which he has named *solanine*. This new vegetable basis, when pure, is in the state of a perfectly white powder, resembling somewhat the cholesterine in

* COLLECTANEA MEDICA, Art. 3.

† *Journal de Pharmacie*, August 1821.

‡ *Op. cit.* September 1821.

its aspect. It belongs to the class of alkalies, and forms an indistinct compound, not crystallizable with different acids. Its bitter taste is more fully developed by the acetic than by any other acid. It possesses highly narcotic powers on the animal economy, producing great somnolency, the evacuation of much glary and mucous matter from the intestines, and sickness at the stomach.* Recent experiments by Mr. VOGEL have not only confirmed the fact, that the expressed oil of bitter almonds owed its poisoning quality to the presence of prussic acid; but have led also to the mode of completely freeing that oil from the obnoxious principle in question: notwithstanding which circumstance, Mr. Vogel has found that the oil continued to possess highly deleterious properties, which seem essentially inherent to the oil itself, and independent of the hydrocyanic acid.†

Various statements have been given, by different authors, of the action of water on magnesia; the sum of which, however, goes to consider that earth as insoluble in water. Dr. FYFE, a lecturer on chemistry in Edinburgh, has, however, proved, by direct experiments, that magnesia is soluble in water; that, like lime, it is more soluble in cold than boiling water, (a singular fact, for which we are indebted to Dalton;) and that the quantity of that earth taken up by the former, when compared to that dissolved by the latter, is as 5760 to 36000.‡

Under the head of Pharmacy, it will not be improper to mention the publication of a work of some value, on the best methods of detecting the adulteration of drugs and other pharmaceutical articles, whether simple or compound, by Professor EBERMAYER; lately translated from the German into French, by Dr. KAPELER and Mr. CAVENTOU, of Paris. §

### IX. NATURAL HISTORY, VEGETABLE PHYSIOLOGY, AND BOTANY.

We shall notice but few subjects in this department of our Essay.

The remarks of Dr. MACCULLOCH on marine luminous animals present many curious and interesting points, which naturalists had hitherto improperly neglected. From the observations of this gentleman, there can be no doubt of the phosphorescence of sea-water, when agitated in a dark night, being owing to the presence of marine animals. ‖

Mr. JAMESON has communicated to Dr. BREWSTER some observations on the hatching of fowls from eggs which have

---

* *Journal de Pharmacie*, September 1821. † *Loc. cit.* October 1821.

‡ *Edinburgh Philosophical Journal*, October 1821.

§ *Manuel des Pharmaciens et des Droguistes.* 2 vols. 8vo. Paris, 1821.

‖ *Quarterly Journal of Science*, No. xxii.

been laid subsequent to the death of the male bird. The fact had hitherto been controverted; but Mr. Jameson's experiments have now put it beyond a doubt.*

The idea of Mr. HUMBOLDT's geographical distribution of vegetables, has probably been the means of suggesting a similar arrangement, with regard to insects, to Mr. LATREILLE, one of the best entomologists in Europe.† The observation seems to teach us that the countries the most abundant in animals with articulated feet, more especially insects, are those of which the vegetation is the richest, and the most rapidly renewed. We have already mentioned the remarks made by Mr. MACAIRE on the *lampyris noctiluca* and *splendidula*, in another place. It results from his inquiries, that the phosphorescent matter of that insect is composed principally of albumen.‡

The late summer has been remarkable for the number of large and strange fishes which have been thrown upon the coast of Scotland, particularly that washed by the Irish Sea. Perhaps, the most singular of these is the sword-fish, (the *xiphias gladius* of Linnæus,) which was thrown on the coast of Kirkbean. It measured ten feet in length, and four and a half feet round the thicker part of the body. The sword, or rostrum, which is the most interesting part of this singular animal, measures three feet eight inches in length.§

The existence of the unicorn seems to be now put beyond doubt, by the report of Mr. CAMPBELL, the missionary, who brought the head of one of those animals to Europe.‖ The account of the formidable leech of Ceylon, by Dr. DAVY, will be perused with interest.¶

The tree which produces the caoutchou, or elastic gum, has again been described by Mr. BRINGIER, who found it growing in the region of the Mississipi, on the Askensas and Red River.** A plant, which dissolves in water, has been mentioned in our last Number. It is found in the South of France, in the form of a green and membranaceous envelope filled with a species of jelly, containing a number of elongated filaments.

Dr. YULE has published an account of the Zealand flax (*phormium tenax*), which he had read before the Royal Society of Edinburgh. The fibres of the leaves are of greater strength than those of flax or hemp; and the islanders of the South Sea, as the late Sir JOSEPH BANKS informed us, apply this plant to many valuable purposes.††

---

* *Edinburgh Philosophical Journal*, No. x.

† *Annales du Museum d'Histoire Naturelle*, vol. iii.

‡ *Biblioth. Universelle*, May 1821.

§ *Edinburgh Philosophical Journal*, No. x.

‖ *London Med. and Phys. Journal*, Oct. 1821.

¶ *Op. cit.* Dec. 1821.

** *Edinburgh Philosophical Journal*, No. x.

†† *Op. cit.* No. x.

A remarkable plant, of the order fungi, has been found growing in a solution of submuriate of ammonia. Dr. FLEMING has communicated an account of this curious discovery to Dr. Brewster; and has accompanied his description with a drawing of the plant itself.*

In the garden at Arundel Castle, the seat of the Duke of Norfolk, several aged fig-trees are growing as standards, and seldom fail to produce ripe fruit. The largest measures six feet nine inches in circumference, at two feet from the surface of the ground: it there divides into two large arms, each of which is eight feet and a half in circumference; and the extended branches of the tree cover a space of thirty feet in diameter.†

Mr. DAVID DON, the keeper of the Lambertian Herbarium, is about to publish a Prodromus Floræ Nepalensis. In this work many new plants will be made known; not a few of them originally discovered by Dr. Francis Buchanan Hamilton.

Mr. SAUSSURE, some time ago, announced that the alkalies and saline substances, met with in vegetables, were derived from the soil in which they were planted. Messrs. SCHRADER and BRACONNOT, who repeated many of the experiments of Saussure, and contrived others, came precisely to a contrary conclusion;—namely, that the greater part of the saline principles to be found in vegetables were the produce of vegetation, and were not absorbed by the plants from the soil. Mr. LASSAIGNE, to set this question at rest, planted several seeds in powder of sulphur, which were kept moist with distilled water. The grains germinated, and the plants grew to the height of six centimetres, surmounted by several leaves. On analyzing these, after incineration, the same proportion of phosphate and carbonate of lime were found in them which were given by the same weight of the ungerminated grains.‡

More accurate descriptions of the varieties of cicuta have been published by Dr. CHEVALLIER, than those which are to be found in the generality of works on botany. We beg the attention of editors of Dispensatories and Pharmacopœiæ to this interesting subject.§

A second edition of the new Flora of the environs of Paris has been published by Dr. MERAT; of which the report made upon it to the Pharmaceutical Society speaks highly. The present edition contains the Cryptogamia.|| Nor is the work of Mr. D'AVRIGNY, on Medical Botany, to be passed over in silence, because it appears to be written with the humble pretension of assisting the junior part of the profession in acquir-

---

* *Edinburgh Philosophical Journal*, No. ix.

† *Transactions of the Horticultural Society of London*, vol. iv. p. 2.

‡ *Journal de Pharmacie*, Nov. 1821. § *Op. cit.* Oct. 1821.

|| *Nouvelle Flore des Environs de Paris.* 2 vol. 2d edition. 1821.

ing a sufficient knowledge of a branch of medical science, which is generally too much neglected.*

---

Numerous as the topics are which we have reviewed in the present Essay, there are others on which, but for the narrowness of our limits, we should have equally bestowed a portion of our attention. On cutaneous diseases,—on the history of medicine,—on medical ethics,—and on veterinary medicine and surgery, we have said nothing. This is a voluntary omission on our part, as no deficiency of materials for observation exists in any of those departments; but we conceived that, in describing the progress of medical science during the last six months, it might be deemed superfluous in us to enumerate more than the really important facts belonging to the principal branches of medical knowledge. How far we have succeeded in accomplishing the latter object, our readers will be enabled to judge from the perusal of the preceding pages. As chroniclers, it was our duty to narrate, without intruding reflections. If, on one or two occasions, we have suffered ourselves to be led away, either by our regard for the importance of the subject, or by our contempt for the futility of certain pretensions, we trust that our general impartiality will suggest, in the minds of our readers, no other motives for such conduct, on our part, than zeal for the profession, and an anxious desire to afford every information in our power to the supporters of the London Medical and Physical Journal. Marked by no brilliant discovery,—neither conspicuous for any extraordinary fact,—the medical EPOCH we have been recording will yet be found to have been fruitful in many curious and important results.

A. B. G.

---

* *Principes de Botanique Medicale, contenant l'Abregé de l'Anatomie et de la Physiologie Vegetale, l'Enumeration et la Description des Plantes Medicamenteuse, &c. &c.* Par A. E. C. LOEUILLARD D'AVRIGNY, D.M. Paris, 1821.

